# Prion Protein Expression Regulates Embryonic Stem Cell Pluripotency and Differentiation

**DOI:** 10.1371/journal.pone.0018422

**Published:** 2011-04-04

**Authors:** Alberto Miranda, Eva Pericuesta, Miguel Ángel Ramírez, Alfonso Gutierrez-Adan

**Affiliations:** 1 Departamento de Reproducción Animal y Conservación de Recursos Zoogenéticos, Instituto Nacional de Investigación y Tecnología Agraria y Alimentaria, Madrid, Spain; 2 Centro de Investigación en Sanidad Animal, Instituto Nacional de Investigación y Tecnología Agraria y Alimentaria, Madrid, Spain; Instituto de Medicina Molecular, Portugal

## Abstract

Cellular prion protein (PRNP) is a glycoprotein involved in the pathogenesis of transmissible spongiform encephalopathies (TSEs). Although the physiological function of PRNP is largely unknown, its key role in prion infection has been extensively documented. This study examines the functionality of PRNP during the course of embryoid body (EB) differentiation in mouse *Prnp*-null (KO) and WT embryonic stem cell (ESC) lines. The first feature observed was a new population of EBs that only appeared in the KO line after 5 days of differentiation. These EBs were characterized by their expression of several primordial germ cell (PGC) markers until Day 13. In a comparative mRNA expression analysis of genes playing an important developmental role during ESC differentiation to EBs, *Prnp* was found to participate in the transcription of a key pluripotency marker such as *Nanog*. A clear switching off of this gene on Day 5 was observed in the KO line as opposed to the WT line, in which maximum *Prnp* and *Nanog* mRNA levels appeared at this time. Using a specific antibody against PRNP to block PRNP pathways, reduced *Nanog* expression was confirmed in the WT line. In addition, antibody-mediated inhibition of ITGB5 (integrin αvβ5) in the KO line rescued the low expression of *Nanog* on Day 5, suggesting the regulation of *Nanog* transcription by *Prnp* via this *Itgb5*. mRNA expression analysis of the PRNP-related proteins PRND (Doppel) and SPRN (Shadoo), whose PRNP function is known to be redundant, revealed their incapacity to compensate for the absence of PRNP during early ESC differentiation. Our findings provide strong evidence for a relationship between *Prnp* and several key pluripotency genes and attribute *Prnp* a crucial role in regulating self-renewal/differentiation status of ESC, confirming the participation of PRNP during early embryogenesis.

## Introduction

Cellular prion protein (PRNP) is a ubiquitous membrane glycoprotein whose abnormal self-replicating, misfolded form is widely believed to cause several central nervous system disorders, collectively known as Transmissible Spongiform Encephalopathies (TSE) [1]. PRNP is expressed in a broad range of vertebrate tissues such as spleen, lymph nodes, lung, heart, kidney, muscle and mammary glands [2], [3]. It is also found in the gonads (spermatogenic cells and ovaries) [4] but most abundantly occurs in the central nervous system. In embryogenesis, mouse *Prnp* mRNA is first highly expressed, mostly in the differentiating neuroepithelium, between E8.5 and E9, during the transition from anaerobic to oxidative metabolism [5] and thereafter expands to non-neuronal tissues at E13.5 [6]. Coupled to the fact that PRNP is highly conserved across species, these data point to an important role for this protein. However, actual evidences exist that make PRNP an absolute mystery. These evidences are that *Prnp*-knockout mice [5], [7], [8], cattle [9] and goats [10] show no drastically modified developmental phenotype and only display subtle changes such as synaptic transmission abnormalities [11], [12], disturbed morphology [13], some alterations in circadian rhythm [14] and cognitive deficiency [15], [16], [17], suggesting mechanisms able to withstand the absence of the protein. Similar observations were made when this gene was inhibited in adult neurons [18]. To explain these data, it has been hypothesized that another host-encoded protein is able to compensate for the lack of PRNP [19]. The main candidates for this compensating role are the PRNP-related proteins Doppel (PRND) and Shadoo (SPRN). The postembryonic expression of Doppel is restricted to the male testis, though *Prnd* mRNA has been detected during embryogenesis. Curiously, the absence of Doppel was observed not to provoke alterations in embryonic and postnatal development, but its deficiency causes male infertility in mice [20]. In contrast, Shadoo shows overlapping mRNA expression with *Prnp* in the brain and has revealed neuroprotective properties at this site. Shadoo has also been attributed a role in embryogenesis such that its downregulation in a *Prnp*-null embryo invariably gives rise to a lethal phenotype between E8 and E11 [19]. Hence, both these proteins play a crucial role in mammalian embryogenesis and could explain the lack of a severe phenotype in PRNP-knockout mammals as a step towards deciphering the biological role of this protein.

The expression of *Prnp* in embryonic stem cells (ESC) and immortalized cells has also been addressed. Thus, the expression of *Prnp* in mouse ESC is 5 times lower than its expression in the brain, yet is 1.5 times higher than its expression in somatic cells [21]. Moreover, during the immortalization of fibroblast MJ90 cells by *Tert* transfection, *Prnp* expression is almost three times greater [22]. In mouse ESC, *Prnp* is again elevated 1.4 and 6.2 times during the first two weeks of differentiation [23] while during their guided cardiogenic differentiation, the expression of the gene is more than 20-fold higher [24]. Curiously, direct reprogramming of somatic cells (MEFs/B-cells) to a pluripotent state (induced pluripotent stem cells, iPS) increases the expression of *Prnp* up to 27-fold [25]. Recently, several studies have associated PRNP with pluripotency. For example, PRNP has been demonstrated to be a marker of haematopoietic stem cells, supporting their self-renewal [26]. Other data indicate that PRNP may be implicated in the biology of glioblastoma, breast cancer, prostate and gastric cancer [27], [28] or, in other words, PRNP is involved in long term proliferation as are stem cells. In addition, it has been reported that the absence of *Prnp* significantly slows the regeneration process of acutely damaged hind-limb tibialis anterior muscles of mice compared to wild-type muscles, affecting the myogenic precursor cells [29]. Collectively, these findings reveal a physiological function of PRNP in immortalized pluripotent ESC.

Despite advances made to date, the real function of PRNP is still unclear with ascribed roles in neuroprotection, the response to oxidative stress, cell proliferation and differentiation, synaptic function and signal transduction [30]. The study of the physiological functions of PRNP seems to be key to understanding both prion diseases and its possible implications in development and cancer biology. Given the widely described similarity of embryoid bodies (EBs) and early postimplantation embryos, we selected EBs as an ideal model [31]. Herein, by comparing stem cell differentiation to EBs in *Prnp*-KO and WT mouse cell lines, we identify the first non-redundant function of PRNP in ESC differentiation during early embryogenesis along with a relationship between the expression of *Prnp* and that of several key pluripotency markers.

## Materials and Methods

### Embryo Collection

Mice were kept on a 14-h light/10-h dark cycle. 10-week-old 129/OLA *Prnp* knock-out female mice were superovulated by intraperitoneal injections of 10 IU of pregnant mare serum gonadotropin (PMSG) (Folligon; Intervet, Boxmeer, Holland) followed 48 hours later by 10 IU of hCG (Chorulon; Intervet). On the day of hCG injection designated Day 0, the female mice were paired with males of the same strain to allow mating. After disinfecting with 70% alcohol and opening the abdominal wall, the oviducts were excised by clamping, dissecting the peritoneum and fat between the ovary and tube, and cutting the whole oviduct from the proximal end. After washing and flushing the oviduct from the proximal end by incising the whole oviduct with a 30-gauge needle, two-cell embryos were selected by 100x microscopy. All the animals were kept in an animal house and handled using procedures and protocols approved by the Animal Care and Ethical Committee (Informe CEEA 2009/009) of the *Instituto Nacional de Investigación y Tecnología Agraria y Alimentaria* (INIA, Madrid), guidelines of the European Union (Directive 86/609/EEC) and current Spanish regulations (BOE 252/34367-91, 2005).

### Production of ESC and EBs

For ESC production [32], two-cell embryos were collected 1.5 days after hCG injection and placed in a droplet of tempered KSOM-LIF medium under oil in a 5% CO_2_ atmosphere at 37°C. After 48 h in KSOM-LIF, the *in vitro* cultured blastocysts were plated individually in 96-well plates coated with 0.1% gelatine and containing mitomycin-C (Sigma-Aldrich corporation St. Louis, MO, USA) treated mouse embryonic fibroblasts (MEF). They were cultured in ESC medium composed of Dulbecco's modified Eagle medium (DMEM plus 4500 mg/l glucose, glutaMAX, and pyruvate; Invitrogen, Carlsbad, CA, USA) supplemented with 20% FBS (PAA Laboratories Cölbe Germany), 2 mM glutamine, 1 mM MEM nonessential amino acids solution, 1 mM β-mercaptoethanol, 1000 U/ml LIF (leukemia inhibitory factor), and an antibiotic mixture containing 100 U/ml penicillin and 100 µg/ml streptomycin. The blastocysts were allowed to attach to the MEF, until expansion without any further experimental interference for four days. After that time, cell clumps originating from the blastocysts were trypsinized and pipetted directly into a well of a 96-well plate containing MEFs and ESC medium. Approximately 4 days after trypsinization, compact cell colonies resembling ESC colony morphology could be detected and these cells were trypsinized in a 24-well plate containing MEFs and ESC medium. In this process, undifferentiated ESCs were separated from differentiated ones and from old MEF, and also expanded in number. Clones that were not confluent were replated on the same plate. When trypsinizing cells from a 35-mm dish for the first time for a particular clone, half the cells were frozen at −80°C in foetal calf serum with 10% DMSO media. This was considered the first passage. For cell line expansion, cells were trypsinized at 80% of confluency.

For embryoid body (EB) differentiation [33], ESC colonies were digested with 0.05% trypsin-EDTA. The dissociated cells were collected in EB medium (DMEM plus (4500 mg/l glucose, glutaMAX, and pyruvate) supplemented with 20% FBS, 2 mM glutamine, 1 mM MEM nonessential amino acids solution, 1 mM β-mercaptoethanol, and an antibiotic mixture containing 100 U/ml penicillin and 100 µg/ml streptomycin and plated on a gelatine-coated dish for 45 min to allow the MEF cells to adhere. Non-adherent cells were collected and plated onto non adherent plastic Petri dishes in the same EB medium. The medium was replaced every 2 days.

### RNA Extraction, Reverse Transcription, and mRNA Transcript Quantification

RNA was prepared from ESC or EBs of each experimental group (WT or *Prnp* KO) using the *ULTRASPEC total RNA Isolation Reagent Kit* (BIOTECX laboratories, INC) according to the manufacturer's instructions. Immediately after extraction, the RT reaction was carried out following the manufacturer's instructions (Promega, Madrid, Spain) to produce cDNA. Tubes were heated to 70°C for 5 min to denature the secondary RNA structure, allowing Random Primer and Oligo dT attachment, and then the RT mix was completed with the addition of 5 units of Superscript RT enzyme. The tubes were then incubated at room temperature for 10 min and next at 42°C for 60 min to allow the reverse transcription of RNA, followed by 70°C for 10 min to denature the RT enzyme. To detect each transcript, we used 2 µl of the cDNA sample in the RT-PCR.

mRNA transcripts were quantified by real-time qRT-PCR [34]. At least three replicate PCR experiments were conducted for all the genes of interest. Experiments were designed to contrast relative levels of each transcript and histone H2az in each sample. PCR was performed by adding a 2- µl aliquot of each sample to the PCR mix (*Quantimix Easy Sig Kit*, Biotools) containing the specific primers. Primer sequences, annealing temperatures, and the approximate sizes of the amplified fragments of all transcripts are provided in Table 1. The comparative cycle threshold (CT) method was used to quantify expression levels (*User Bulletin, Abi Prism 7700 Sequence Detection System*, Applied Biosystems). Quantification was normalized to the endogenous control H2az. Fluorescence was acquired in each cycle to determine the threshold cycle or the cycle during the log-linear phase of the reaction at which fluorescence increased above background for each sample. Within this region of the amplification curve, a difference of one cycle is equivalent to doubling the amplified PCR product. According to the comparative CT method, the CT value was determined by subtracting the H2az CT value for each sample from the CT value of each gene in the sample. CT was calculated using the highest sample CT value (i.e., the sample with the lowest target expression) as an arbitrary constant to be subtracted from all other CT sample values. Fold changes in the relative gene expression of the target were determined using the formula 2^–CT^.

**Table 1:** Primers used for RT-PCR.

Gene	Primer sequence 5′->3′	Size	GenBank
*Prnp*	GATTATGGGTACCCCCTCCTTGG	289	NM_011170.2
	ATGGCGAACCTTGGCTACTGGC		
*Nanog*	AGGGTCTGCTACTGAGATGCTCTG	363	M6047
	CAACCACTGGTTTTTCTGCCACCG		
*Pouf51 (Oct3/4)*	GGAGAGGTGAAACCGTCCCTAGG	391	M17031
	AGAGGAGGTTCCCTCTGAGTTGC		
*Genesis (Foxd3)*	TCTTACATCGCGCTCATCAC	171	M4758
	TCTTGACGAAGCAGTCGTTG		
*Stat3*	CAGAAAGTGTCCTACAAGGGCG	340	U06922
	CGTTGTTAGACTCCTCCATGTTC		
*Mapk1*	CCTTCAGAGCACTCCAGAAAGT	74	NM_011949.3
	ACAACACCAAAAAGGCATCC		
*Gp130*	ATTTGTGTGCTGAAGGAGGC	186	M83336
	AAAGGACAGGATGTTGCAGG		
*Itgb3*	GTCACATTGGCACCGACAACC	556	NM_016780.2
	CCACACTCAAAAGTCCCGTTC		
*Itgb5*	GGCTGCTGTCTGCAAGGAG	511	M96614
	TCAAAGGATGCCGTGTCC		
*Itga6*	GTGAGGTGTGTGAACATCAG	540	NM_008397.3
	CATGGTATCGGGGAATGCT		
*Shadoo*	TTGGCCTGTACAAAGTTGAG	186	NM_183147.2
	CTGCAATGAGGGAAAAGCCT		
*Prnd (Doppel)*	GCTCCAAGCTTCAGAGGCCACAGTAGCA	580	M1346999
	TTACTTCACAATGAACCAAACGAAAC		
*Sod1*	GGGATTGCGCAGTAAACATTC	67	NM_011434.1
	AATGGTTTGAGGGTAGCAGATGA		
*Slc2a1*	CCAGCTGGGAATCGTCGTT	76	M23384
	CAAGTCTGCATTGCCCATGAT		
*Irs2*	CTCTGACTATATGAACCTG	339	NM_001081212.1
	ACCTTCTGGCTTTGGAGGTG		
*Gapdh*	ACCCAGAAGACTGTGGATGG	247	BC102589
	ATGCCTGCTTCACCACCTTC		
*T (Brachyury)*	GCTGTGACTGCCTACCAGCAGAATG	220	M913
	GAGAGAGAGCGAGCCTCCAAAC		
*Hnf3-β*	GGACGTAAAGGAAGGGACTCCAC	174	M938
	AGCCCATTTGAATAATCAGCTCAC		
*Nestin*	AGTGTGAAGGCAAAGATAGC	316	M23742
	TCTGTCAGGATTGGGATGGG		
*H2AZ*	AGGACGACTAGCCATGGACGTGTG	212	NM_016750
	CCACCACCAGCAATTGTAGCCTTG		

### Statistical Analysis

Data were analyzed using the *SigmaStat* (Jandel Scientific, San Rafael, CA) package by one-way repeated-measures ANOVA with arcsine data transformation.

### Semiquantitative PCR

cDNA was obtained as described above and PCR was performed with a PCR mix (*Go taq Flexi DNA polymerase Kit*, Promega) following the manufacturer's instructions. The PCR products were analyzed on a 2% agarose gel by ethidium bromide staining. The gel was visualized under ultraviolet light to identify the specific band for each gene analyzed.

### Antibody Inhibition Experiments

EBs of both ESC lines were re-suspended on Day 5 in EB medium alone or supplemented with SHA31 anti-mouse PRNP antibody (CISA, INIA), with anti-rabbit ITGB5 antibody (ABCAM) or control antibody (mouse IgG) at final concentrations in the medium of 0.2 µg/ml. The EBs were collected 6 or 24 hours later and frozen for mRNA extraction.

### Terminal Deoxynucleotidyl Transferase-Mediated dUTP Nick End-Labelling (TUNEL) Assay

For the TUNEL method of detecting apoptosis, EBs cultured for 5 days were fixed in 4% paraformaldehyde in PBS, pH = 7.4, for 1 h, and then washed in PBS/1%BSA three times. The fixed EBs were permeabilized with 0.1% Triton X-100 in PBS for 15 min at 37°C and then washed again in PBS/1%BSA three times. TUNEL assays were performed using the *In Situ Cell Death Detection Kit*, *TMR Red* (Roche) according to the manufacturer's protocol. As control, it was checked that a lack of TdT in the TUNEL mix completely abolished labelling.

## Results

### Two distinct types of *Prnp*-KO EBs exist

**Figure 1:**
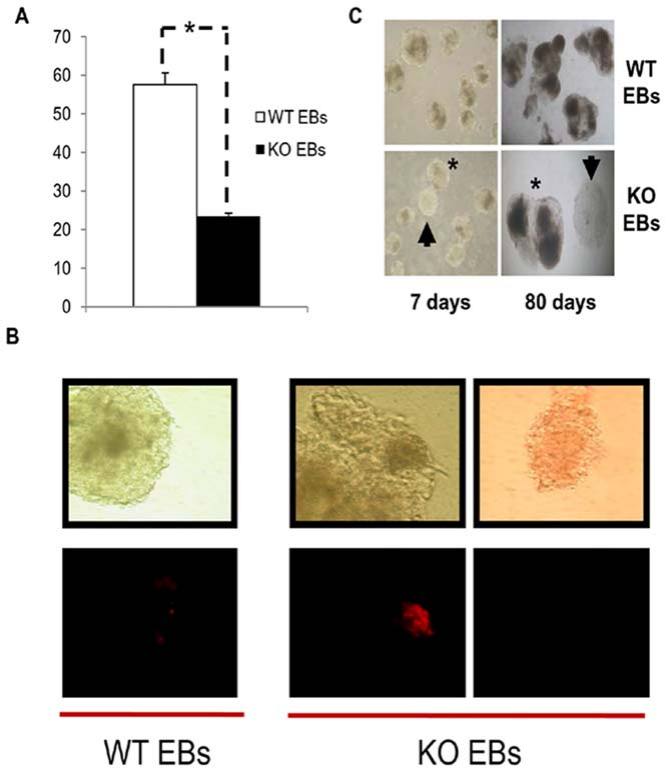
Apoptotic and macroscopic effects produced by the delection of *Prnp*. A) Comparing the numbers of EBs in the WT and KO lines (* P<0.05. Error bars, s.e.m.; “Y” axis represents “number of EBs per plate”) B) TUNEL analysis of wild type (WT) and *Prnp*-null (KO) embryoid bodies (EBs). C) Morphology of EBs on Day 7 and Day 80 of differentiation. The KO ESC line produced 2 types of EBs: one similar to WT (*) and the other called PGC-*like* KO EBs (arrowheads).

Two lines of *Prnp*-KO ESC were produced in a 129Ola background (that of the WT control line R1). Since both these lines develop in the same way, the results for each line were collectively considered. Confluent WT and KO ESC lines were trypsinized and grown in a LIF-free medium without the feeder layer to form EBs. From Days 1 to 3, large numbers of unclustered cells could be seen among the KO EBs. This could be the reason of a significantly reduced EB numbers on Day 5 (Fig. 1A). TUNEL apoptosis analysis on Day 5 of ESC differentiation revealed a low number of isolated cells positive for immunostaining in the WT line (Fig. 1B) (2–5% of the WT EB surface). Extensive apoptotic red zones were detected in the KO EBs on Day 5 of differentiation (10–15% of the KO EB surface), which could explain the high number of floating cells observed in the early EB culture supernatants. Interestingly, two types of EBs were observed on Day 5 in the KO group. About 40% of the EBs were white showing no apoptosis signal, and 60% were morphologically similar to the WT EBs. The white EBs persisted after a long period of differentiation (more than 80 days) while in the WT line, only a small number of EBs looked white after 5 days of differentiation (1–2%) and these disappeared over the following 2 days (Fig 1C). In addition, after 30 days of differentiation, mature EBs in both KO populations were plated on a feeder layer in complete stem cell medium (including LIF and bFGF), and after 3 passages, we could only recover cells positive for alkaline phosphatase of the white KO population. mRNA was extracted from the different types of EB and it was noted that Gp130 and Stat3 (proteins involved in maintaining pluripotency) were upregulated only in the white KO population (data not shown).

### *Prnp*–KO ESC and EBs express different pluripotent markers to the WT line during differentiation

**Figure 2:**
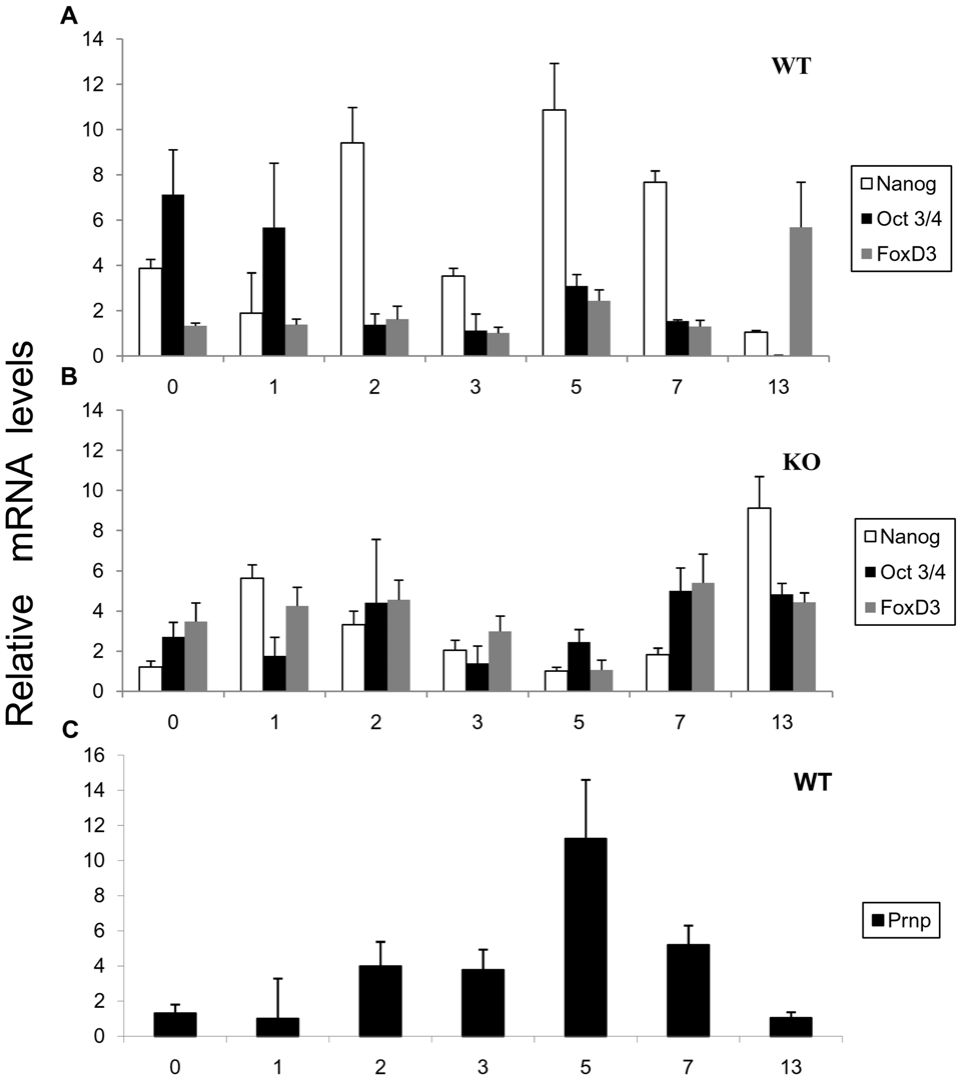
Pluripotency in the WT and *Prnp*-null cell lines. WT EB mRNA (**A**) and KO EB mRNA (**B**) expression patterns of three pluripotency genes during differentiation. Note the lack in the KO line (**B**) of an increase in *Nanog* expression on Day 5. C) WT EB mRNA expression pattern of *Prnp* during differentiation (Days 0, 1, 2, 3, 5, 7 and 13). *Prnp* is expressed at its highest levels on Day 5 (Error bars, s.e.m.).

The mRNA expression of pluripotency markers was examined in both the WT and *Prnp*–KO lines. We selected the genes *Oct3/4* (*Pou5f1*), *Nanog*, and *FoxD3* (*Genesis*) because the feedback regulating cycle of these genes is among the most important for maintaining stem cell pluripotency [35]. Stem cells from the WT line showed significantly higher *Nanog* and *Oct3/4* and lower *FoxD3* transcript levels than KO ESC (Fig. 2A and 2B), indicating a different background of pluripotency in the KO and WT lines.

In order to better understand the role of PRNP in differentiation, *Prnp* mRNA was quantified from the ESC stage (Day 0) to Day 13 EBs. Stem cell lines were cultured in ESC medium without LIF and samples were taken on Days 0, 1, 2, 3, 5, 7 and 13. Next, mRNA was extracted and transcripts were quantified by qRT-PCR. During WT EBs differentiation, *Prnp* showed increasing levels of expression starting on Day 2 until Day 7, showing peak expression on Day 5 (Fig. 2C). In the WT line, no significant differences could be detected in mRNA levels of *FoxD3* until Day 13 when an expression increase was observed (Fig. 2A). However, *Prnp* expression was negatively correlated with that of the pluripotency marker *Oct-4*. Thus, higher *Oct-4* expression levels were detected on Days 0 and 1 followed by reduced expression until Day 13, following an opposite expression pattern to *Prnp* and confirming that ES cells had indeed differentiated. *Nanog* was also downregulated on Day 13, accordingly with the differentiation status. Interestingly, *Nanog* expression showed a similar pattern to *Prnp,* with increasing expression levels observed from Day 2 to Day 7 peaking on Day 5 (Fig. 2A). In contrast, in the KO line, *Oct-4* and *FoxD3* expression levels showed some variation during early differentiation but high mRNA levels persisted on Days 7 and 13 (Fig. 2B). Further, *Nanog* expression levels were highest on Day 13, indicating the pluripotent state of these cells even after 13 days of differentiation (Fig. 2B).

**Figure 3:**
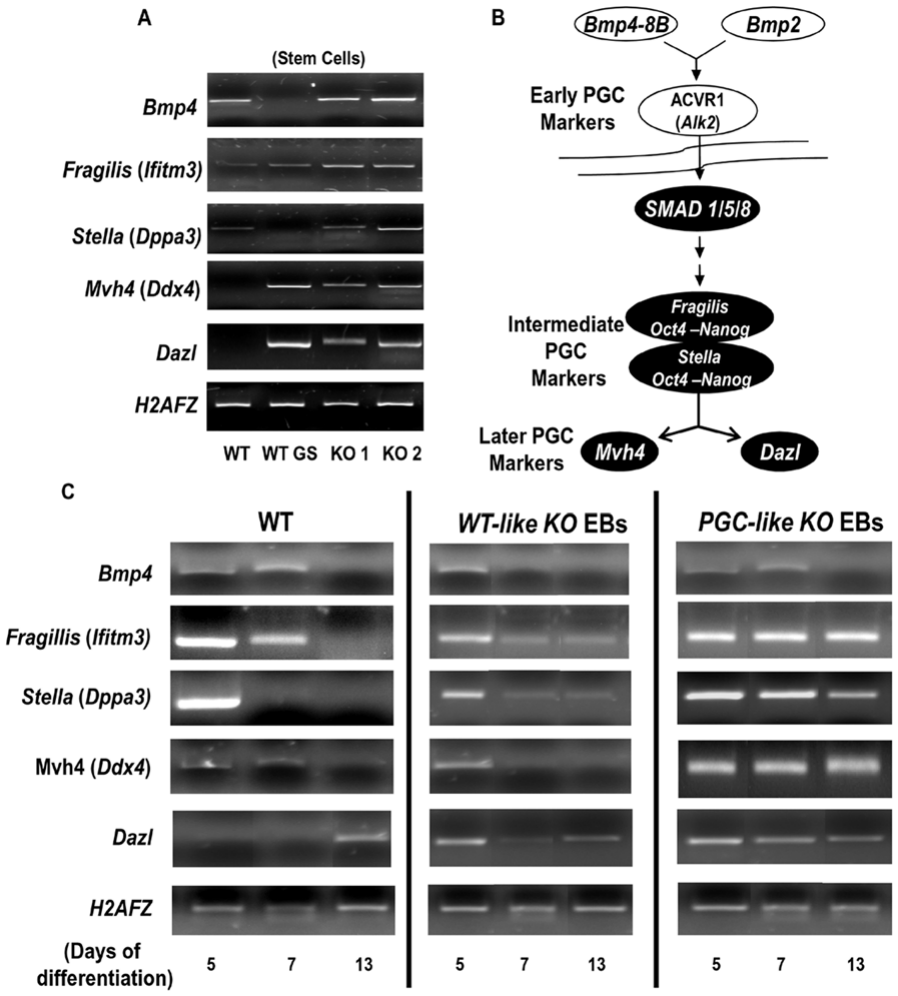
Consequences of *Prnp* absence in the BMP pathway. A) Detection of PGC markers (*Bmp4, Fragillis, Stella, Mvh4 and Dazl*) in WT ESC, WT GS and the two different *Prnp* KO ESC lines (KO 1 and KO 2) by PCR. Knock out cells show all the PGC markers analyzed in stem cell state. B) Diagram representing the BMP pathway showing the early, intermediate and later PGC markers (figure adapted from Young et al. 2009). C) Detection of primordial germ cell markers (*Bmp4, Fragillis, Stella, Mvh4 and Dazl*) by PCR during differentiation in WT EBs, WT-*like* KO EBs and PCG-*like* KO EBs.

To understand this initial developmental status of the different pluripotency markers in the KO ESC, we examined the expression of bone morphogenetic protein (BMP) genes, which are related to the germ cell program. These genes were chosen because KO EBs show characteristics comparable to those of germ cells, as the only cells in the organism that repress their somatic differentiation program in favour of a germline-specific network of RNA regulation. Indeed, BMPs regulate the differentiation of PGCs and are also able to potentiate pluripotency in the presence of LIF. Our semiquantitative PCR revealed increased expression levels of all the PGC markers in the KO stem cells, regardless of the stem cell line (Fig. 3A). The expression of two later PGC markers, *Mvh4* (*Ddx4*) and *Dazl* (Fig. 3B), was lacking in the WT line but was detected in WT cells grown in a GS medium and in the KO lines.

When we assessed the same markers during the time course of EB differentiation, it was found that early (*Bmp4*) and intermediate [*Fragillis* (*Ifitm3*) and *Stella* (*Dppa3*)] PGC markers (Fig. 3B) were upregulated on Day 5 in WT EBs and that these were gradually replaced with the later expressed *Mvh4* and *Dazl* from Day 7 to 13 (Fig. 3C). The same occurred in normal-appearance KO EBs, but in the white EB population all these markers persisted at all the time points. For this reason, we called the white EBs derived from the KO ES cells *PGC-like* to differentiate them from the KO EBs of similar morphological appearance to WT EBs (designated *WT-like* KO EBs).

### Characterizing the WT and *Prnp*-KO lines during differentiation to EBs

**Figure 4:**
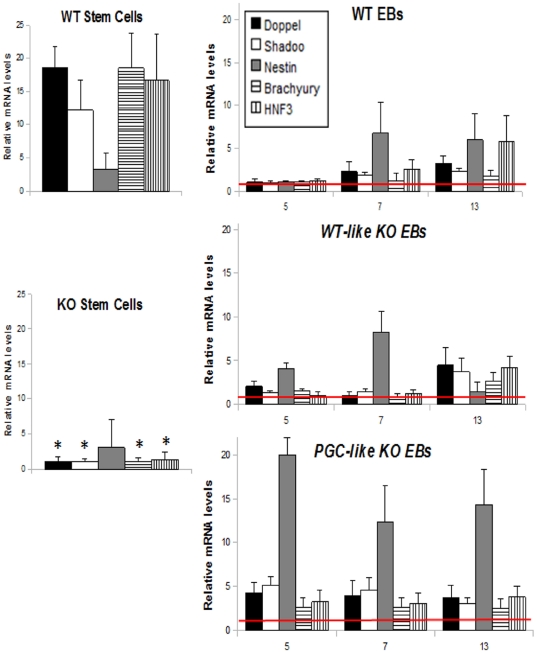
Differentiation transcription behaviour of the WT and *Prnp*-null cell lines. mRNA expression patterns of PRNP related proteins (Doppel and Shadoo) and early markers of endoderm (*Hnf3*), mesoderm (*Brachyury -T-*) and ectoderm (*Nestin*) in WT and KO stem cells and in WT, *WT-like* KO and *PGC-like* KO EBs during differentiation (Days 5, 7 and 13). (* indicates statistical differences for the transcription of each gene between WT and KO ESC at P<0.05) (Error bars, s.e.m).

**Figure 5:**
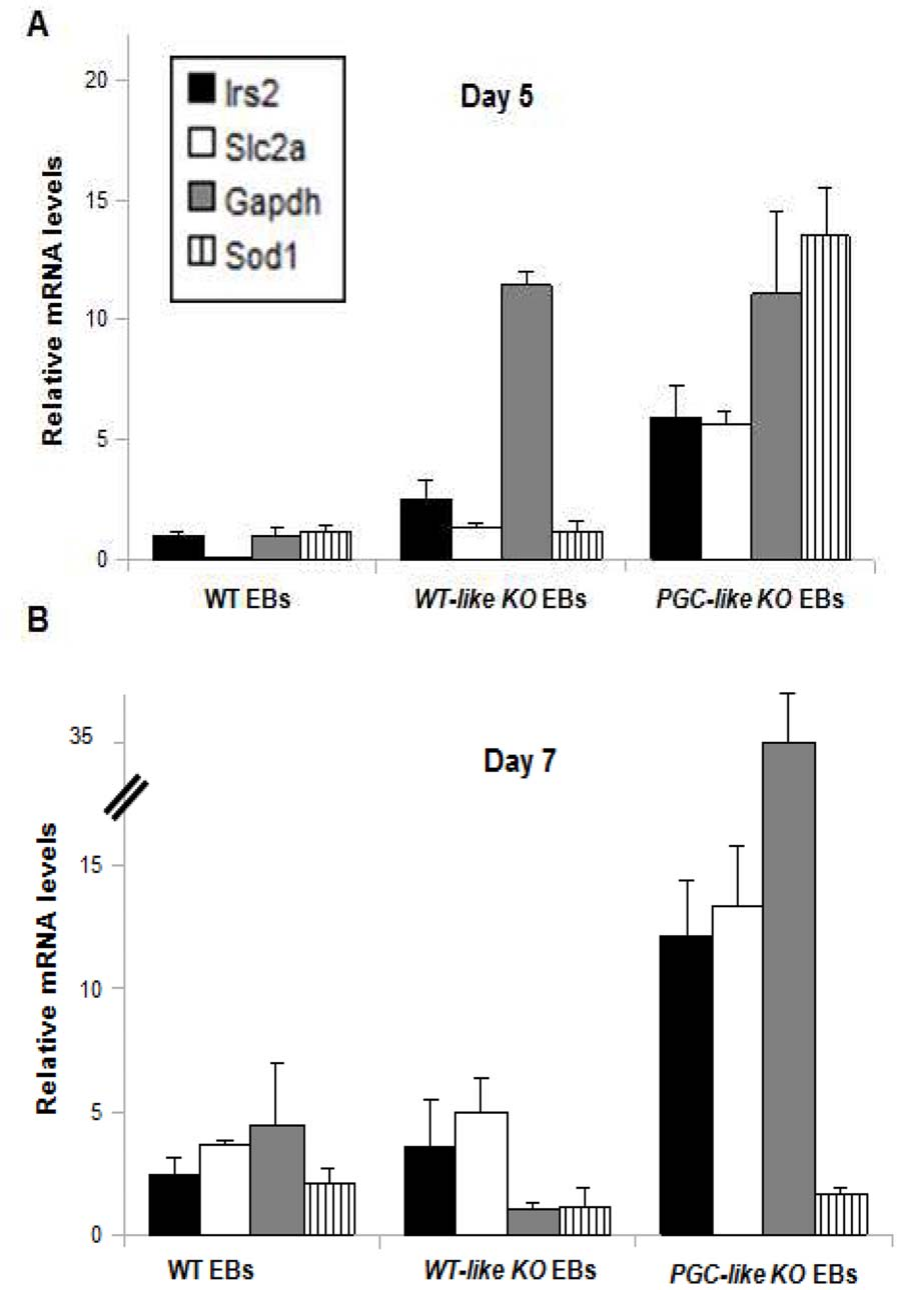
Metabolic transcription behaviour of the WT and *Prnp*-null cell lines. WT, *WT-like* KO EB and *PGC-like* KO EB mRNA expression patterns for *Irs2* (Insulin Receptor Substrate 2), *Slc2a* (Glucose Transporter 1 - *Glut1* -), *Gapdh* (Glyceraldehyde 3-phosphate Dehydrogenase) and *Sod1* (Superoxide Dismutase 1) on Days 5 (A) and 7 (B). (Error bars, s.e.m.).

The appearance of two EB populations in the KO line could be the consequence of two different ways of compensation and expression of *Shadoo* and *Doppel*, two genes whose functions are redundant to those of *Prnp* in some tissues and developmental stages. Accordingly, we then examined mRNA levels of these genes during the time course of EB differentiation. In addition, to determine if the differences between the three types of EBs were due to differences in tissue differentiation, we analyzed the mRNA expression of early markers of ectoderm (*Nestin*), mesoderm (*Brachury* -T-) and endoderm (*Hnf3*) (Fig. 4). We considered it might be also interesting to determine the metabolic state of these EB populations since PRNP is thought to be involved in protection from oxidative stress [30]. Thus, we examined mRNA levels of glucose capturing genes (*Slc2a* and *Irs2*) and glycolysis genes (*Gapdh*) (Fig. 5) giving information about oxidative metabolism, and mRNA levels of the SOD1 gene (*Sod1*), which codes for a protein whose antioxidant activity is regulated by PRNP [36], [37].

WT ESC expressed all the transcripts except Nestin in higher amounts than the KO ESC (Fig. 4, Day 0). Interestingly, during differentiation, mRNA levels of all markers were significantly raised and maintained from Day 5 in the *PGC-like* KO EBs (Fig. 4) compared to WT and *WT-like* KO EBs, which showed similar expression among each other. However, in the *WT-like* KO EBs the absence of PRNP resulted in non-expression of *Nestin* on Day 13 (Fig 4) suggesting a role for PRNP in neural cell differentiation during early embryogenesis. This phenomenon was not observed in the *PGC-like* KO EBs since these cells maintained similar transcription levels during the differentiation time examined.

On the other hand, the mRNAs of *Irs2, Slc2a, Gapdh* and *Sod1* were augmented in the *PGC-like* KO EBs on Days 5 and 7 (Fig. 5). *Gapdh* mRNA was also elevated on Day 5 in *WT-like* KO EBs but reached baseline levels on Day 7, suggesting a transition feature. This metabolic situation was maintained until Day 13 in both kinds of KO EB (data not shown) and indicates enhanced glycolytic and oxidative metabolism in the *PGC-like* KO EBs.

### *Stat3* mRNA is upregulated in the later stages of differentiation

**Figure 6:**
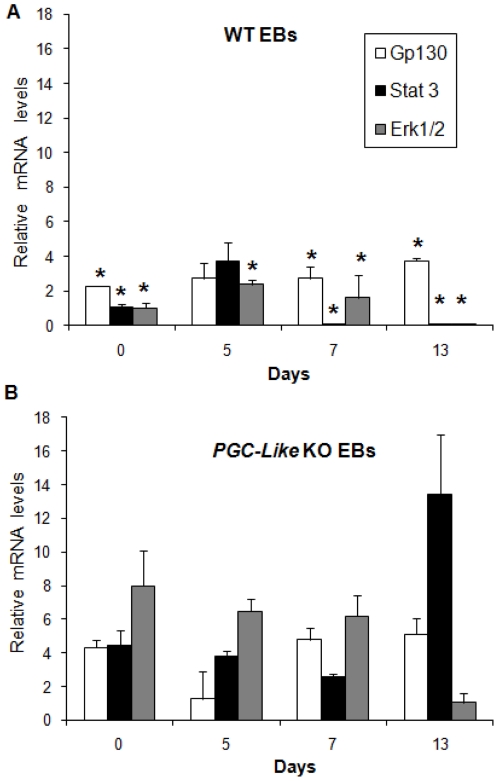
LIF pathway regulation in the WT and *Prnp*-null cell lines. WT EB mRNA (**A**) and *PGC-like* KO EB mRNA (**B**) expression patterns of some LIF pathway genes during differentiation (Days 0 [(Stem Cell], 5, 7 and 13). (*indicates significant differences for a particular gene between WT and *PGC-like* KO EBs (P<0.05)) (Error bars, s.e.m.).

Our experimental protocol includes the use of LIF-starved ES medium, so the receptor (*Gp130*) and main effector genes (*Erk1/2* and *Stat3*) of the LIF pathway were analysed. ERK 1/2 is known to be involved in the loss of pluripotency and is also a downstream component of the PRNP biochemical matrix, while STAT3 contributes to the maintenance of self-renewal. WT EBs showed lower expression of *Erk1/2* mRNA than *PGC-like* KO EBs, disappearing on Day 13 (Fig. 6). The same lower levels were observed for *Stat3* in the WT EBs, but disappearing on day 7. However, in the *PGC-like* KO EBs, increased transcription of this gene was observed on Day 13, indicating and supporting the previously described pluripotent state of these EBs. In contrast, Gp130 expression failed to vary or only showed subtle variations during the time course of differentiation, indicating that the upregulation of *Stat3* transcription is not related to activation of the LIF pathway.

### *Prnp* regulates *Nanog* mRNA expression

**Figure 7:**
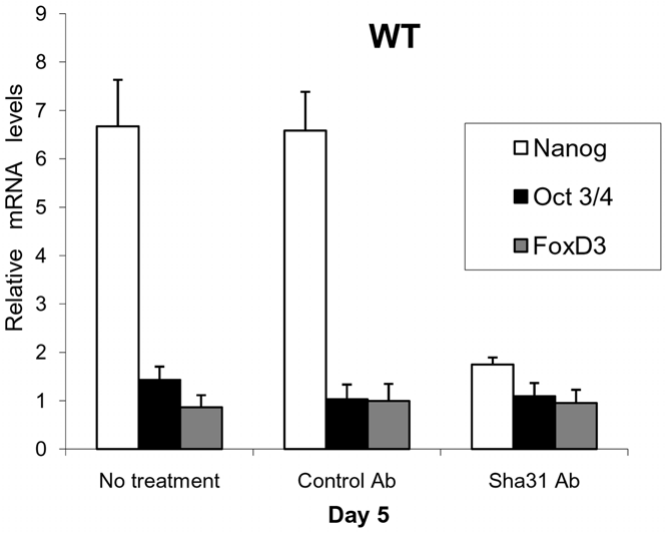
Blocking of PRNP by the monoclonal antibody Sha31. On Day 5 and after 6 hours of antibody treatment, it is revealed that *Nanog* mRNA transcription is PRNP-dependent during differentiation and independent of feedback from the pluripotency cluster genes *Oct3/4* and *FoxD3*. (Error bars, s.e.m.).

On Day 5 of EB differentiation, the highest levels of *Prnp* and *Nanog* were detected in the WT EBs. Similar waves in their mRNA expression pattern were also observed suggesting *Prnp*-dependent *Nanog* transcription (Fig. 2A and Fig. 2C). To confirm this relationship, we conducted *Prnp* inhibition experiments. Several studies have shown that the addition of specific PRNP antibody to the culture medium inactivates this protein's signal transduction pathways [38], [39] so we proceeded to add the monoclonal antibody Sha31 to WT EBs on Day 5. Interestingly, a significant reduction in *Nanog* mRNA levels was detected, even over the shorter time interval (6 h) (Fig. 7), and this effect persisted for 24 hours without refreshing the antibody (data not shown). Further, mRNA expression patterns of important PRNP related markers on Day 5 (e.g. integrins αvβ5 and α6 and the metabolic gene *Sod1*), which were differentially expressed between the *PGC-like* KO EBs and WT EBs, became similar (data not shown). This inhibition of *Nanog* expression confirms the involvement of *Prnp* during EB differentiation.

### *Nanog* expression on Day 5 is blocked by integrin beta5 in *Prnp*-KO EBs

**Figure 8:**
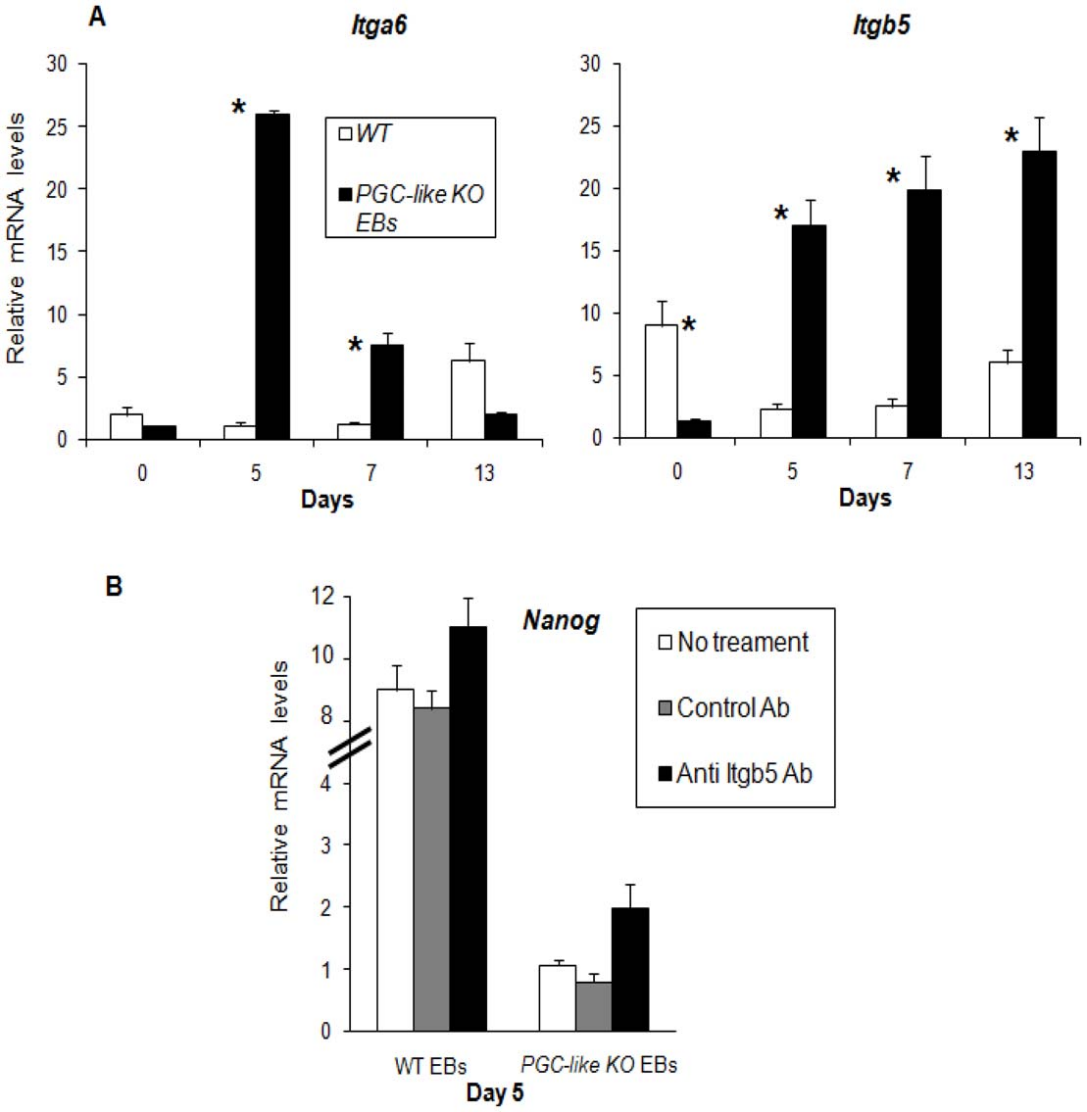
Influence of *Prnp* in the integrin matrix. A) Detection of PRNP-related integrins (*Itga6* and *Itgb5*) in WT and KO stem cells on Day 0, and in WT EBs and *PGC-like* KO EBs on Days 5, 7 and 13. B) Blocking of ITGB5 by a polyclonal antibody shows that *Nanog* mRNA transcription is ITGB5-dependent during differentiation (*indicates significant differences between WT and *PGC-like* KO EBs on a particular day (P<0.05)) (Error bars, s.e.m.).

Integrins are essential for PGC functionality [40], [41] and it has been reported that the absence of *Prnp* may be compensated by these proteins [42], [43]. Since integrin signalling is crucial in promoting mouse ESC self-renewal [44], we speculated that *Prnp* expression might also be related to this integrin expression during ESC differentiation. So we examined by real time PCR the transcription levels of three genes coding for integrins αvβ3 (*Itgb3*), αvβ5 (*Itgb5*), and α6 (*Itga6*) in stem cells and during early EB differentiation (Days 5, 7, and 13). We found that *Itgb3* was only detected in *PGC-like KO* EBs on Day 5. Similar transcription levels of *Itga6* were found in the KO stem cells and in the WT line. During differentiation, significantly elevated expression was observed on Day 5 in the *Prnp*-KO background population; this transcription gradually fell from Day 7 to 13. In the WT, expression was increased only on Day 13 (Fig. 8A). Interestingly, mRNA for *Itgb5*, described to be a gonadal tissue marker [41], appeared in higher amounts in the WT ESC than in the KO ESC; however, expression was higher in the *PGC-like KO* EBs at all the differentiation times (Fig. 8A).

Because the expression of *Itgb5* on Day 5 differed significantly between WT and *KO*, we designed an *Itgb5* antibody mediated inhibition experiment to analyse its effect on the transcription patterns observed on Day 5 of EB differentiation. Surprisingly, when ITGB5 was blocked at the membrane level in the *PGC-like KO* EBs, *Nanog* mRNA transcription was significantly augmented, while in the WT line, this treatment had no significant effect (Fig. 8B). These results indicate that in the absence of *Prnp*, the expression of *Nanog* on Day 5 of EB differentiation may be partially mediated by the inhibition of this integrin.

### Correlated *Prnp* and *Nanog* expression in WT mice points to a role for both genes in the development of the foetal gonads

**Figure 9:**
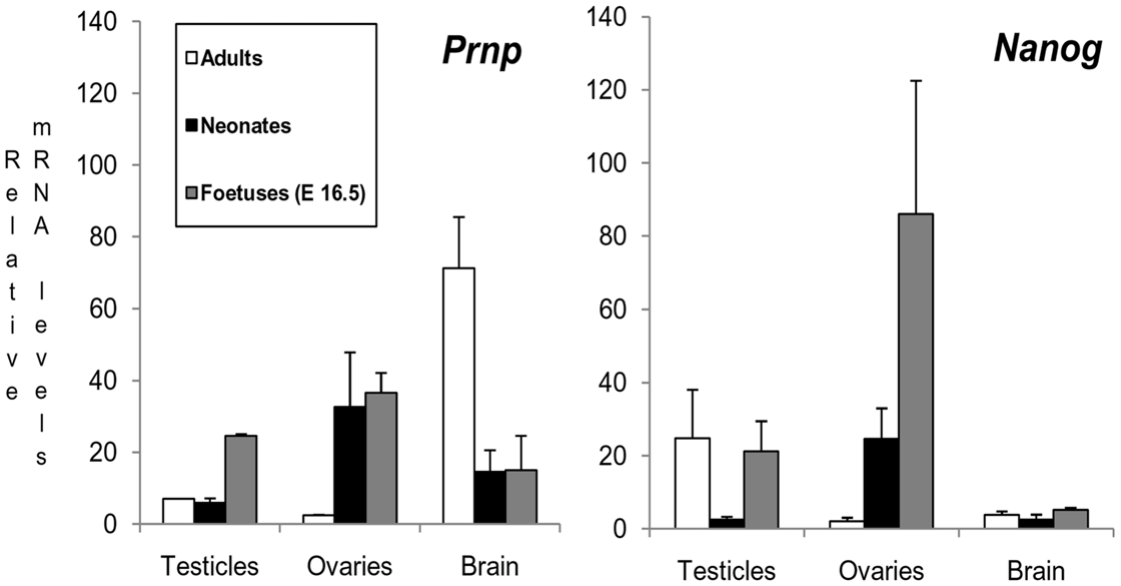
*Prnp* and *Nanog* expression in testicles, ovaries and brain in adult, neonates and foetal mice at embryonic day 16.5 (E16.5). Note that correlative *Prnp-Nanog* expression only occurs in gonadal tissues. (Error bars, s.e.m.).

We quantified the in vivo transcription of *Prnp* and *Nanog* in the brains, testicles and ovaries of E16.5 WT mouse foetuses, neonates and adults. These tissues were selected because *Prnp*-KO ESC and their derived EBs showed an ectodermal gene expression pattern. In brain, as expected, *Prnp* was highly expressed, with no *Nanog* expression detected in any case (Fig. 9). In the testicles, a clear increase in *Prnp* mRNA levels was seen during the foetal period; and in the ovaries, this was observed in the foetal and neonatal period. *Nanog* expression in these two tissues was upregulated at the same time points as *Prnp* except in adult testicles, where *Nanog* showed clear upregulation contrary to the behaviour of *Prnp*. However, *Doppel* was upregulated at this point (data not shown) and could explain the absence of *Prnp* expression in the adult testicle. These results indicate that correlative *Nanog-Prnp* mRNA expression, possibly due to *Nanog Prnp*-dependant transcription as was seen in the “*in vitro*” experiments, occurs in the gonads but not in neuronal tissues.

## Discussion

The key role of prion protein in infection has been expansively reported, yet its physiological function remains elusive. To address this issue, we designed a comparative analysis during differentiation of WT and *Prnp*-KO ESC. In the current study, we employed ESC to assess the potential role of PRNP in early embryo development, examining how this locus becomes transcriptionally activated when potential transient compensatory mechanisms are still not established. We here present the first evidence that *Prnp* is involved in regulating the self-renewal/differentiation status of stem cells. Its expression was found to affect that of several pluripotent genes, including *Nanog,* a protein that drives constitutive stem cell self-renewal and safeguards pluripotency [45], [46] through its involvement in the Sox2-Oct3/4 transcription factor network [47], [48], [49], [50], [51].

The main pathway through which ESC-derived cells become differentiated cells involves the formation of embryoid bodies, thus denoted because of their similarity to the early postimplantation embryo. Embryoid bodies are formed “*in vitro*” after several days of growth of ESC in the absence of LIF (leukemia inhibitory factor). These bodies are the final ESC population that has the potential to give rise to all the cell types of adult tissues and have been described as the developmental equivalent of the egg cylinder-stage mouse embryo, with an outer endodermal layer and a core of differentiating cells, often comprised of epithelial-lined cavities [52]. Hence the data obtained in this work may represent what happens “*in vivo*” during embryo development.

Our results indicate that despite the widespread expression of key genes of pluripotency in WT and *Prnp*-KO ESC lines (e.g., Oct4) and their characteristic undifferentiated cell morphology, functional ESC may represent only a small fraction of the total number of cells grown under self-renewal conditions. Effectively, in our KO ESC, a large *PGC-like* stem cell population was identified. Further, mRNA levels of early endoderm and mesoderm markers were significantly lower, while early neuronal ectoderm marker mRNA levels were fairly similar to those in WT ESC. Taken together with the fact that PGC markers were also expressed, an *ectodermal* stem cell type was defined in the KO ESC, according to the simple absence of *Prnp*. Indeed, a heterogeneity in the stem cell population leading to different transcription behavior has been previously described based on *Nanog* mRNA levels [53]. This could explain the appearance of two different kinds of EB when LIF was removed from the culture medium in the KO line, based on the detected absence of *Nanog* transcription in this EB population. Thus, phenotypically “undifferentiated” cells may consist of a heterogeneous population of functionally distinct cell types, and small changes in gene expression could lead to the predominance of one of these types over the others.

Interestingly, in *Prnp*-KO ESC, *Doppel* and/or *Shadoo* were clearly downregulated. However, on Day 5, *PGC-like KO* EBs overexpressed Doppel and Shadoo, showing the altered expression of early embryo layer markers and also enhanced glucose and oxidative metabolism. This last feature has been related to a high rate of proliferation in cancer or stem cells (Warburg effect) [54], [55] and to increased protection against oxidative stress, according to the high pluripotency level of these *PGC-like KO* EBs and to compensation for the high sensitivity to oxidants of the KO lines, respectively. Until now, Doppel and Shadoo, ascribed to the PRNP family, were known to compensate for the absence of PRNP, having redundant (in the case of Shadoo in brain [19]) or exclusive (in the case of Doppel in male testicles [20]) functions. However, we noted that *WT-like KO* EBs exhibited similar expression patterns of these genes with subtle differences in comparison to WT EBs. Thus, we could speculate that an ESC, lacking *Prnp* yet showing increased *Doppel* and *Shadoo* gene expression during differentiation, will give rise to a living system closely resembling PGCs, morphologically different to WT cells, more metabolically active in terms of differentiation [55] and with a high degree of pluripotency. Conversely, if Doppel and Shadoo were not overexpressed, KO EBs would be practically identical to their WT counterparts. Our results therefore suggest that the absence of PRNP is not counteracted by the presence of Doppel and Shadoo, as previously thought. The two different populations of KO EBs with a completely different expression pattern for these genes, further supports the idea of an irreplaceable role of PRNP in this process, most likely in coordinating the expression of indispensable proteins for the appropriate behaviour of the stem cells.

Recently, it has been indicated that during certain embryogenesis processes some redundancy for PRNP interactions resides within the integrin pathway [43], and that integrin signalling is crucial for promoting mouse ESC self-renewal [44]. Here, we analysed the expression of three integrins during PRNP-null stem cell differentiation to investigate if these integrins play some sort of role in maintaining the stemness of these ESC. Integrin αvβ3 (ITGB3) and integrin αvβ5 (ITGB5) are expressed in mouse foetal developing gonads (from Days 10.5 to 13.5); ITGB3 is specific to primordial germ cells [41] and ITGB5 plays important roles in maintaining stemness in undifferentiated mouse ESC [56]. The observation of *Itgb3* transcription only on Day 5 in the KO EB population indicated primordial germ cell transcription activity at this stage. Moreover, the higher expression of *Itgb5* on Days 5, 7 and 13 was in agreement with the presence of the primordial germ cell markers identified in the *PGC-like KO* EBs. It has been reported that integrin-α6 (ITGA6) function is required for the early stages of lens cell differentiation [57]. The higher expression of *Itga6* we identified on Day 5 in KO EBs, and on Day 13 in the WT EBs, could point to the cell differentiation activity of PGCs in the KO EBs, and of the three embryonic layers in the WT line.

Our results also indicate a relationship between PRNP and integrins in ES cells and during differentiation. It has been shown that PRNP binds some extra cellular matrix (ECM) proteins [43], [58], [59], and other important ECM proteins, such as integrins, could also interact or cooperate with PRNP in this process. Integrins have been the focus of several studies over the past few years, not only because of their capacity to bind ECM, but also because of their ability to activate a number of cell signaling pathways, including those of differentiation and pluripotency [44], [56]. It is also known that *Prnp* downregulates *Itgb3* mRNA expression in Mesenchymal Embryonic Cells (MEC) [42], highlighting the importance of integrins in the PRNP biochemical matrix. Other authors have determined that PGCs contain subpopulations showing low or null integrin-α6 (ITGA6) expression with a greater ability to develop into pluripotent stem cells [40]. The fact that several authors have also related the upregulation of certain integrin transcription with a loss of pluripotency and self-renewal [44], suggest a pivotal link between PRNP or integrins and these processes. Our results revealed that *Itga6*, *Itgb3* and *Itgb5* were deregulated in the KO line. Specifically, *Itgb3* (data not shown) and *Itgb5* mRNAs appeared in high quantities in the *PGC-like KO* EBs on Day 5 and, only for the last gene, this population of EBs was labelled by this integrin for longer times (Days 7 and 13). Since, as mentioned earlier, these integrins are confirmed PGC and gonadal tissue markers, respectively, this suggests the idea of arrested EBs in that ectoderm cell line, also consistent with the loss of self-renewal in a situation of high integrin expression [44].

In addition, the expression of *Erk1/2*, previously incriminated in differentiation, was significantly downregulated on Day 13 in the *PGC-like KO* EBs, at the same time an increase in *Stat3* transcription and downregulation of *Itga6* were observed. These results suggest that *PGC-like KO* EBs seemed to acquire pluripotent and PGC self-renewal characteristics on Day 13. Further, in our antibody-mediated inhibition experiments, when ITGB5 was blocked on Day 5 at the membrane level in the *PGC-like KO* EBs, the important pluripotency mRNA transcript *Nanog* was significantly elevated, while this treatment had no effect in the WT line, though a non-significant upward trend was observed. Similar results were reported when mouse ESC cultured on laminin or fibronectin (activators of integrin pathways) in ESF7 medium showed low immunostaining for alkaline phosphatase, weak immunostaining in immunocytochemical experiments detecting NANOG and a low population of NANOG positive cells in flow cytometric analysis [44]. However, when the mouse ESC was treated with an anti-β1 integrin antibody (blocking the interaction ECM-integrins) these findings were reversed [44].

We also observed here that the disruption of PRNP by knocking out through transgenesis or knocking down through antibody inhibition led to diminished *Nanog* mRNA levels (especially on Day 5, when peak *Prnp* and *Nanog* were observed in the WT line), accompanied by high levels of *Itgb5* mRNA. These data thus indicate the possible *Prnp* regulation of *Nanog* transcription via *Itgb5* during early differentiation to EBs. Moreover, the results of our “*in vivo*” experiments suggest that this relationship during early differentiation between *Prnp* and *Nanog* specifically occurs in gonadal but not in brain tissue. In summary, our data point to the first known relationship between PRNP and NANOG, one of the most important proteins needed to maintain pluripotency, with a major role in the self-renewal of ESC and their differentiation into gonadal cells.

A loss of PRNP functionality was largely proposed as one of the main causes of a prion disease physiopathology. There is a lack of a *premortem* way of detecting them and bearing in mind that *Nanog* levels remain low or disappear during the first stages of differentiation of *Prnp*-KO ESC, the use of this gene as a marker of prion disease and, more importantly, as an early detection method could be investigated. Otherwise, PRNP is not essential for ESC survival since other pathways could support its absence, although a significant percentage of cells die in the first stages of differentiation to EBs. In contrast, *Prnp* knockout or downregulation modifies the normal macroscopic and transcriptional behaviour of the stem cell during differentiation, confirming the participation of PRNP in early embryogenesis [19]. The association between *Prnp* and pluripotency marker expression also provides evidence of this contribution of *Prnp* to stem cell differentiation (e.g. the role of PRNP in neural cell differentiation [60]). This feature is mediated by *Nanog* and not compensated by the PRNP-related proteins Doppel and Shadoo, suggesting for the first time a non-redundant function of PRNP in maintaining pluripotency and differentiation during early embryogenesis.
